# A Case of Steroid-Induced Gastric Perforation in a Ten-Year-Old Child

**DOI:** 10.7759/cureus.51780

**Published:** 2024-01-07

**Authors:** Brajendra N Mishra, Dhirendra Kumar, Gaurav Mishra

**Affiliations:** 1 General Surgery, Tata Steel Hospital, Noamundi, IND; 2 Emergency Department, Tata Steel Hospital, Noamundi, IND

**Keywords:** clinical disorder, omental patch, uncommon surgical emergency, gastric ulcer, anterior gastric perforation

## Abstract

*Gastric perforation* is a rare yet critical clinical disorder that demands prompt medical attention. Gastric ulcers often manifest on the anterior wall of the stomach, underscoring the importance of early detection for an improved prognosis. This study delves into a specific case, shedding light on a 10-year-old male child diagnosed with steroid-induced gastric perforation. The diagnosis was established through a meticulous examination of the clinical history and a plain abdominal X-ray, culminating in a timely and decisive surgical intervention for repair.

## Introduction

Gastric ulcers (GUs) are commonly seen due to helicobacter pylori, NSAID digestion, or rarely in Zollinger-Ellinson syndrome. At times, these ulcers may even lead to perforations, which is a more severe condition. The method of an omental patch for treating perforated ulcers was initially described by Cellan-Jones (1929) and introduced by Graham in 1937 [1,2]. Perforation is the second most and rare clinical complication of gastric ulcer [2]. An anterior hole in the pyloro-duodenal region is the most prevalent form of perforation connected to the stomach [3]. Depending upon the location of the perforation, patient clinical presentation varies. The development of GU depends on several factors such as alcohol consumption, usage of non-steroidal anti-inflammatory drugs (NSAIDs), smoking, Helicobacter pylori infection, pelvic fracture, trauma, inflammation, etc [4].

In this study, we discuss the case of a 10-year-old male child who suffered from a steroid-induced anterior gastric antral perforation. This case is noteworthy due to the rarity of its occurrence in the pediatric population.

## Case presentation

A ten-year-old male child weighing 24 Kg was referred to the Department of Surgery, Tata Steel Hospital in Jharkhand, India. The patient was admitted after encountering increasing pain in his epigastric region. He revealed pain in the abdomen associated with nausea and postprandial vomiting.

He did not have any previous history of any type of gastritis or peptic ulcer disease. His vitals at the time of the visit were temperature of 101˚F, respiratory rate of 22 breaths per minute, blood pressure of 90/60 mmHg, and heart rate of 116 beats per minute. He was dehydrated, and his skin was pale yellow with a toxic look. The abdomen was slightly distended and diffusely painful, with stiffness and guarding all over the abdomen. His urine analysis was normal during the examination. Before admission to the hospital, the patient took three doses of prednisolone, each of 10 milligrams (mg), to treat bronchitis.

**Figure 1:**
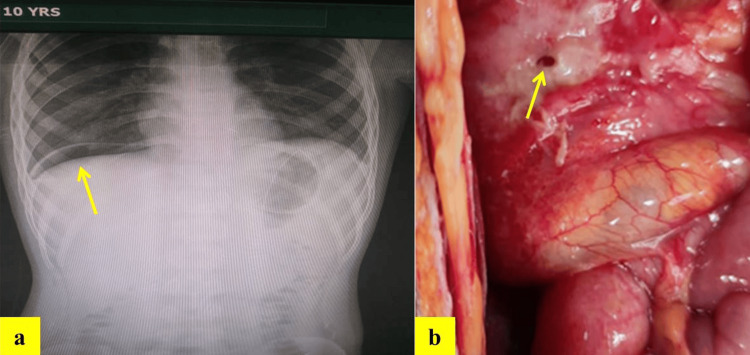
Arrow indicating (a) Gas under right diaphragm and (b) Anterior gastric perforation.

The complete blood count test and serum electrolytes showed hemoglobin of 11.2 gram/deciliter, platelets count of 176,000 per microliter, blood urea of 21 milligrams per deciliter, sodium 132.9 milli-equivalents per liter, potassium 4.01 milli-equivalents per liter, serum creatinine 0.40 milligrams per deciliter, and chlorides 96 milli-equivalents per liter. Abdominal X-rays in the upright and supine postures suggest hollow viscous gastrointestinal perforation having air underneath the right hemidiaphragm (Figure 1a).

Nasogastric and urethral catheters were inserted. Broad-spectrum antibiotics, namely piperacillin plus tazobactam (2.25 grams) and metronidazole infusion (50 milliliters; 250 mg) were administered intravenously, and an emergency exploratory laparotomy was performed. The surgical findings included a 5mm × 5mm perforation on the anterior wall of the gastric antrum wall (Figure 1b). Near about 2 liters of bilious fluid was collected from the peritoneal cavity. After refreshing the margins, an omental patch was used to fix the gastric defect. The abdomen was washed with warm, normal saline throughout the process until the returning fluid was clear. Later, the abdomen was drained and closed. Figure 1 shows an X-ray and surgical results, respectively.

On the 3rd post-operative day, the patient was shifted to wards and started on oral fluids based on the institutional protocols. Post-operative antibiotic treatment and proton pump inhibitors were continued for 10 days. No complications were observed. The patient recovered well and was discharged in good physical condition with a follow-up treatment prescription. This treatment included cefuroxime (250 mg) and metronidazole (200 mg) orally for at least 5 days. On follow-up after a month, clinical inspection and examination revealed no symptoms of dehydration, weight loss, leakage, infection, or fever. The patient was pleased with his overall health, as he recovered well.

## Discussion

Globally perforated peptic ulcer is among the most frequent surgical emergencies. Gastric Perforations have an elevated mortality rate (40%) as compared to a duodenal ulcer (10%). The omental patch is an effective and useful surgical treatment for gastric antral perforation. However, it may not be accessible in uncommon situations such as deep perforations, previous omental surgery, and inflammation [5]. High doses of steroids and antibiotics inhibit the biosynthesis of gastric alkaline responses and enzyme activity inside the stomach walls and may lead to biochemical dysfunction, gastric perforation, and lesions [6].

 Moreover, as the peritoneum completely covers the stomach, perforation of the peritoneum creates a link between the gastric lumen and the peritoneal cavity. If the perforation occurs suddenly, there is no stage for an inflammatory reaction. Therefore, gastric secretions and acids are free to pass through the peritoneal cavity, resulting in chemical peritonitis. Wineni and Setiawan reported a case of a 5-day-old newborn with stomach perforation who underwent primary surgical repair [7].

Laboratory studies, such as blood reports and plain abdominal X-rays, are performed to aid diagnosis. The air beneath the diaphragm, along with pneumoscrotum, ascites, or even the tip of an oro- or nasogastric tube, extends beyond the gastric outline structures [8,9]. The initial management includes the use of broad-spectrum antibiotics in case of infection, supportive care, gastric acid suppression therapy, and primary peritoneal drainage to enhance clinical outcomes before the surgery [10]. No histopathological investigation and bacterial culture were implemented in this study, as no complications or indications were seen.

## Conclusions

Gastric perforation is found to be a very infrequent disease in a child. The location of ulcers within the stomach affects the surgical procedure. They may appear with a smaller sac abscess, commonly linked with peritonitis or retroperitoneal abscess. Gastric perforation in children is a serious and fatal disorder, especially in those who are underweight. Early diagnosis, basic care, primary management, stabilization, nutrient diet, and surgical exploration are all part of initial management.
